# Comparative analysis of phytochemicals and antioxidant activities in seeds and sprouts of different varieties of radish (*Raphanus sativus* L.): TOPSIS-entropy weight method

**DOI:** 10.3389/fpls.2025.1531570

**Published:** 2025-02-07

**Authors:** Caihui Guo, Yi Zhu

**Affiliations:** College of Food Science and Nutritional Engineering, China Agricultural University, Beijing, China

**Keywords:** radish seeds, radish sprouts, antioxidant activity, glucosinolates, phytochemicals, TOPSIS-entropy weight

## Abstract

Many bioactive components in plants are beneficial to health, and their contents in seeds and sprouts are much higher than those in mature parts. This study intended to uncover alterations in nutritional compositions of radish seeds following germination. It also aimed to evaluate the health-promoting potential of both radish (*Raphanus sativus* L.) seeds and sprouts by quantifying representative bioactive compounds and antioxidants across six varieties. The ultimate goal was to identify the optimal radish variety with these beneficial properties through the TOPSIS-entropy weight method. This study measured chlorophyll, carotenoids, anthocyanins, glucosinolates (GLSs), total phenol (TP), vitamin C content, and antioxidant capacities (POD: Peroxidase; PAL: Phenylalanine ammonia lyase; T-AOC: Total antioxidant capacity) in seeds and sprouts of radish grew for 7 days. The GLSs content in seeds was 3 to 6-fold higher than that in sprouts; while contents of anthocyanin, sugar, and TP were much higher in radish seeds than those in sprouts. Chlorophyll, carotenoid content, and POD activity were significantly higher in sprouts than in seeds. Combined with the ideal solution similarity ranking preference method (TOPSIS) entropy weight method, M (Man Tang Hong) was the optimal radish variety. Sprouts generally outperformed seeds in terms of overall phytochemical composition and antioxidant capacities, except for M. Therefore, more sprouts are recommended to be consumed in daily life without choosing specific radish variety. In conclusion, this study supports the health-promoting properties of radish based on a comprehensive deciphering of the nutritional profile of radish seeds and sprouts, both of which are good sources of bioactive compounds.

## 1 Introduction

Cruciferous plants are widely cultivated and used around the world, with definite preventive effects on various chronic diseases and cancers documented by different epidemiological and meta-analyses (Li et al., 2022). In addition to the unique glucosinolates (GLSs), different phytochemicals such as polyphenols, carotenoids, and vitamin C (VC) contained in cruciferous vegetables work together as anti-inflammatory and antioxidant agents (Šamec et al., 2017). Vegetables in the germination stage, as sprouts, have gained increasing attention currently from the public, especially health-conscious consumers (Francis et al., 2022). Sprouts may contain 2 to 10-fold more phytochemicals than vegetables in the mature stage (Choe et al., 2018), and they are fast-growing and short-cycle, which can usually be collected and eaten after 5-10 days (Moreno et al., 2006). Also, it has been extensively explored regarding the beneficial health effects of edible seeds (Kakkar et al., 2023). Many authors have studied different varieties of wheat (Chen et al., 2017; Tian et al., 2019), while fewer have studied buckwheat, broccoli, radish, alfalfa, brown rice, etc (Pajak et al., 2014; Thakur et al., 2021; Wunthunyarat et al., 2020). Seeds also accumulate some bioactive substances (*e.g.*, antioxidants, and vitamins) during germination (Liu et al., 2022), but reduced contents of sugars and some special biochemicals, such as GLSs, in radish seeds (Gamba et al., 2021). These findings support that edible seeds and sprouts are of equal importance for research.

*Raphanus sativus* L. (generally termed Radish) is a popular vegetable from the cruciferous family. Consumption of radish sprouts, a product of seed germination, is increasing as they exhibit higher nutrient levels than the matured portion (Manivannan et al., 2019; Gamba et al., 2021). Dried seeds of radish (RS, Raphani Semen, Lai Fu-zi in Chinese), are listed in China Pharmacopoeia, which can be eaten in daily life and used clinically as medicine to treat food indigestion, upper abdominal distension, constipation, panting, and coughing in China with a long history (Jacky, 2023). In India, RS has a therapeutic effect on asthma and other chest diseases (Aruna et al., 2012), revealing excellent anti-inflammatory and antioxidant properties. Furthermore, sprouts and seeds also show a variety of culinary forms, whether served in salads or cooked with other vegetables, etc. Radish seeds may not be as commonly consumed as their sprouts, which can generally be eaten straight, sautéed, served with salads or in soups. Therefore, both sprouts and seeds of radish can be used as a food with health benefits. There have been reports on GLSs, total phenols (TP), vitamin content and antioxidant capacities in some radish sprouts and seeds. But little is known about the mechanism of these compounds and their antioxidant capacities changing throughout the seed germination process. In addition, there is still no consensus about the use of germination conditions and applied assays in most reports. Moreover, compounds and antioxidant activities vary remarkably in seeds grown under different situations (light source and time, varied seed treatments) (Hanlon and Barnes, 2011; Baenas et al., 2012; Kyriacou et al., 2019; Liu et al., 2022; Bowen-Forbes et al., 2023; Tilahun et al., 2023; Šola et al., 2024). It may also be a challenge to compare the research results of different authors and directly draw consistent conclusions. In addition, researchers put more emphasis on sprouts despite existing comparative analyses on uncovering the potential for nutrient production between seeds and sprouts of radish (Baenas et al., 2012). Therefore, more efforts should be put in measuring and validating the content and antioxidant capacity of various compounds in seeds and sprouts under the same germination conditions. Equal importance should be given to the comparison of seeds and sprouts, two major and equally important sources of nutrients.

Obtaining high-quality sprouts and seeds requires consideration of species and variety selection. Different radish varieties show differences in the phytochemical content of seeds and sprouts at varied stages (Hanlon and Barnes, 2011). Screening for eligible radish variety can therefore provide valuable insights to meet consumer nutritional preferences. Sprouts have many advantages over seeds in terms of improved functional quality, such as more obvious accumulation of minerals, phenolic acids, flavonoids, vitamins and other bioactive compounds (Gan et al., 2017; Le et al., 2019). However, the content of GLSs within radish seeds is much higher than in sprouts (Baenas et al., 2012). GLSs are functional components unique to cruciferous vegetables, possessing abundant bioactive activities involving cancer-protective, antioxidant, anti-inflammatory, antidiabetic, neuroprotective, and cholesterol-lowering (Alloggia et al., 2023). It can thus be determined comprehensively that the nutritional function of radish seeds is not necessarily lower than that of sprouts. While there is still no comprehensive study to highlight the nutritional function of radish seeds and sprouts through comparison. A comparison of the chemical compositions in radish seeds and sprouts will also offer useful information for consumers’ decision-making of their consumption according to their needs.

On these basis, there is a lack of systematic comparisons of bioactive compound contents between seeds and sprouts of radish from the perspectives of comparison methods and radish cultivars. This study was designed with several purposes: (1) to investigate changes in nutrient composition of radish seeds after germination; (2) to compare GLS yield, TP content, and antioxidant capacities of seeds and sprouts from six different cultivars of radish; and (3) to comprehensively evaluate the phytochemicals content such as GLS yield and TP to prioritize radish cultivars, seeds, and sprouts to obtain the best radish varieties. Multi-criteria decision making (MCDM) method: the technique of similarity preference ranking of ideal solutions (TOPSIS), is a time-saving convenient tool that works easily by finding solutions based on the distance to positive and negative ideal solutions and then ranking them accordingly (Nandi and Guha, 2023). The entropy weighting method determines index weights by assessing index values. Index with greater weight will occupy a stronger position in the assessment system and offer more information. TOPSIS-entropy weight method allows for the evaluation of the importance of indicators as well as a comprehensive evaluation of the excellence of different varieties. Thus, this study employed this method to screen for the optimal variety of radish.

This study collected and analyzed data on several radish seeds and sprouts variables including GLSs, chlorophyll, carotenoids and anthocyanins content, total and soluble sugars content, VC, TP content and antioxidant capacities. These data were analyzed in a comprehensive and comparative manner to obtain the optimal variety. Through this exploration, we may understand the popularization of radish seeds and sprouts preferably as functional food ingredients.

## 2 Materials and methods

### 2.1 Plants and germination conditions

Six common varieties of radish seeds were commercial varieties locally: Man Tang Hong, M; Qiu Bai Yu, BY; Tian Cui, T; Ying Tao, Y; Da Qing, Q; Da Hong Feng, HF. It has been confirmed that all radish seeds are hand-picked clean and loose by the farmer, and can either be eaten directly or cultivated into sprouts. Seeds of six radish species (M, BY, T, Y, Q, HF) with full grains and no mold were selected separately for repeated rinsing and soaking in distilled water for 10 h. The soaked seeds were evenly sown in 30*20 cm seedling trays lined with four layers of cotton gauze, and then placed in an incubator at 25°C and 75% relative humidity to dark for 2 h. The next step was incubation under a fixed light/darkness cycle of 16 h/8 h and a light intensity of 1,500 lux, with a watering frequency of 50 mL every 6 hours. Sprouts were collected after 7 days of incubation, and the sprouts and soaked seeds were fast-frozen with liquid nitrogen, respectively. These sprouts and soaked seeds were ground and stored at -80°C for testing.

### 2.2 Extraction and quantification of chlorophyll, carotenoids and anthocyanin contents

#### 2.2.1 Chlorophyll *a*, *b*, and carotenoids

The samples were added with 95% ethanol to mix thoroughly for extraction by oscillation for 2~4 h until the samples were colorless. The next steps were centrifugation (12,000 g for 10 min), and absorbance measurements (665, 649, and 470 nm) after supernatant discarding. The 95% ethanol was used as a reference solution by the following formula (Chen and Wang, 2006):


$$Ca=13.95A_{665}-6.88A_{649}$$

$$Cb=24.96A_{649}-7.32A_{665}$$

$$Cxc=1000A_{470}-2.05Ca-114.8Cb/245$$

Ca, Cb and Cxc are concentrations of chlorophyll *a, b* and carotenoids, respectively (mg/L). Ultimately, their contents were calculated as:


$$\begin{array}{c} Chlorophyll\;a\;content\;(\frac{mg}{g}\;FW)\\ =\frac{\left(Ca*Volume\;of\;extraction\;solution\;(ml)*dilution\;factor\right)}{\left(1000*Sample\;fresh\;weight\right)} \end{array}$$

$$\begin{array}{c} Chlorophyll\;b\;content\;(\frac{mg}{g}\;FW)\\ =\frac{\left(Cb*Volume\;of\;extraction\;solution\;(ml)*dilution\;factor\right)}{\left(1000*Sample\;fresh\;weight\right)} \end{array}$$

$$\begin{array}{c} Carotenoid\;content\;(\frac{mg}{g}\;FW)\\ =\frac{\left(Cxc*Volume\;of\;extraction\;solution\;(ml)*dilution\;factor\right)}{\left(1000*Sample\;fresh\;weight\right)} \end{array}$$

#### 2.2.2 Anthocyanin

The samples were dissolved in 1% HCl in methanol solution, extracted by shaking at 4°C for 30 min, and centrifuged at 12,000 g for 10 min at 4°C for absorbance measurement (530 and 657 nm) after the supernatant was left. Using methanol with 1% HCl as a reference solution, anthocyanin content was determined as follows (Li and Zhu, 2018):


$$Anthocyanin\;content(\frac{mg}{g}FW)=A_{530}-(\frac{1}{4}A_{657})$$

### 2.3 Determination of total and soluble sugar content

The two components were measured using the Total Sugar and Soluble Sugar Assay Kit (Nanjing Jiancheng Bioengineering Institute, Nanjing, China) by referring to corresponding manual, respectively expressed as mg/g FW and mg/mL FW.

### 2.4 Determination of VC content

VC content was quantified, with results in μg/mg FW, following the instruction of a VC assay kit (Nanjing Jiancheng Bioengineering Institute, Nanjing, China).

### 2.5 Determination of TP content

TP determination, with gallic acid as a control and expressed as GAE/g FW, was performed as described by previous research with minor modifications (Zhou et al., 2013). Frozen radish seeds or sprouts were dissolved in 50% alcohol, and then subjected to a 30°C water bath for 1.5 h, shaking every 15 min. The supernatant obtained from centrifugation (6,000 g for 15 min) was mixed with Folin-Ciocalteu reagent, and then 20% sodium carbonate solution, fixed with distilled water. The solution was allowed to stand for 2 h at room temperature and protected from light for absorbance measurement (765 nm).

### 2.6 Determination of the content of GLSs

By referring to prior method with minor modifications (Li and Zhu, 2018), the content of GLSs was determined using following steps. Briefly, the prepared sprouts and soaked seeds were dry vaporized in a boiling water bath for 10 min, adding with 1.5 ml of boiling distilled water, and then in a boiling water bath for 30 min. After removing and cooling down, and voluming to 2.5 ml, the next step was centrifugation (12,000 g for 10 min) to get 1 ml of the supernatant. It was added and mixed with 2 mL of 0.15% (w/w) carboxymethylcellulose sodium solution, and then added with 1 ml of 8 mmol/L palladium chloride chromogenic solution for 2 h of placement in a light-proof environment. The results were expressed as μmol/g FW after absorbance measurement at 540 nm on the UV spectrophotometer.

### 2.7 Measurement of antioxidant capacities

Peroxidase (POD), phenylalanine ammonia lyase (PAL), and total antioxidant capacity (T-AOC), different antioxidants enriched in plant species, were measured to indicate the antioxidant capacities. The measurements (U/g FW for all) were completed using PAL, POD, and T-AOC assay kits (Solarbio, Beijing, China), with strict operations as indicated in the manual.

### 2.8 Application of the TOPSIS-entropy weight method

TOPSIS-entropy weight method was used to analyze the phytochemical content and antioxidant capacities of the assays across multiple metrics to determine the optimal variety of radishes, sprouts, or seeds with preferable health-promoting potential. A dozen indicators such as phytochemical content and antioxidant capacities were determined by identifying the distance from positive and negative ideal solutions, and the treatments were ranked to determine the optimal radish variety. The analysis was performed as described by Ansarifar et al. (2015) and Khodaei, Yu et al (Yu et al., 2020; Khodaei et al., 2021).

### 2.9 Statistical analysis

A Duncan’s multiple range test (P < 0.05) was utilized to determine the level of statistical significance after one-way analysis of variance in IBM SPSS Statistics 26.0. All data (means ± standard deviation) were determined independently in triplicate.

## 3 Results

### 3.1 Contents of chlorophyll, carotenoid, and anthocyanin

**Figure 1:**
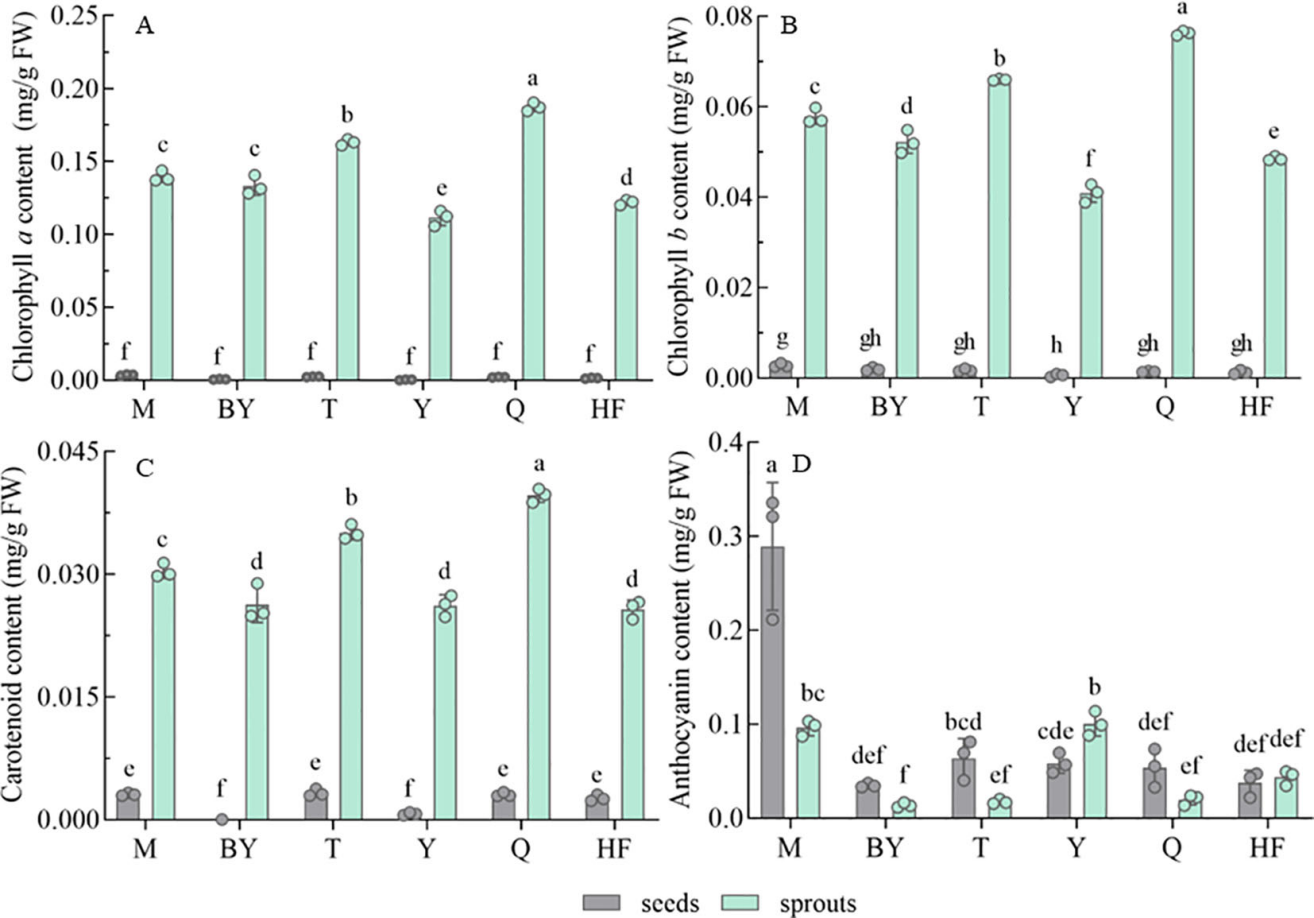
Chlorophyll, carotenoid, and anthocyanin contents of radish seeds and sprouts (germination for 7 days) from different radish varieties. Chlorophyll *a* **(A)**, Chlorophyll *b* **(B)**, Carotenoid **(C)**, Anthocyanin **(D)**. mg/g FW, mg/g FW indicates the comparison of the content of the target substance per gram of seed and per gram of fresh sprout. The data represent mean ± SD values (n = 3). The different lowercase letters indicated significant differences in values (P < 0.05).

Chlorophyll, carotenoids, and anthocyanins were determined in seeds and 7-day-old sprouts of radish from six varieties (**Figure 1**). Significantly higher contents measured from sprouts were observed than from seeds for six radish varieties. At the same weight, the increase ranged from 37.61 to 268.75-fold (chlorophyll *a*, **Figure 1A**), 20.68 to 68.21-fold (chlorophyll *b*, **Figure 1B**), and 9.61 to 36.79-fold (carotenoids, **Figure 1C**). Chlorophyll *a* content in sprouts ranged from 0.112 to 0.187 mg/g FW, chlorophyll *b* from 0.041 to 0.076 mg/g FW, and carotenoids from 0.026 to 0.040 mg/g FW, with significant differences between varieties. Q sprouts had the highest chlorophyll *a*, chlorophyll *b*, and carotenoids, followed by T, while Y had the lowest content. Levels of anthocyanins also varied in the seeds and corresponding sprouts of the six radish varieties (**Figure 1D**). The levels of anthocyanins were significantly higher in the seeds of M and T than in the sprouts (3.00-fold, 3.68-fold). In contrast, levels in Y were significantly higher in the sprouts than in the seeds (1.72-fold), and the differences were not significant in the remaining three varieties. The high-to-low ranks of different varieties based on anthocyanins were M (0.29 mg/g FW), T (0.064 mg/g FW) and BY (0.035 mg/g FW) (for seeds); while Y (0.10 mg/g FW) M (0.096 mg/g FW) and BY (0.01 mg/g FW) (for sprouts).

### 3.2 Total sugars and soluble sugars content

**Figure 2:**
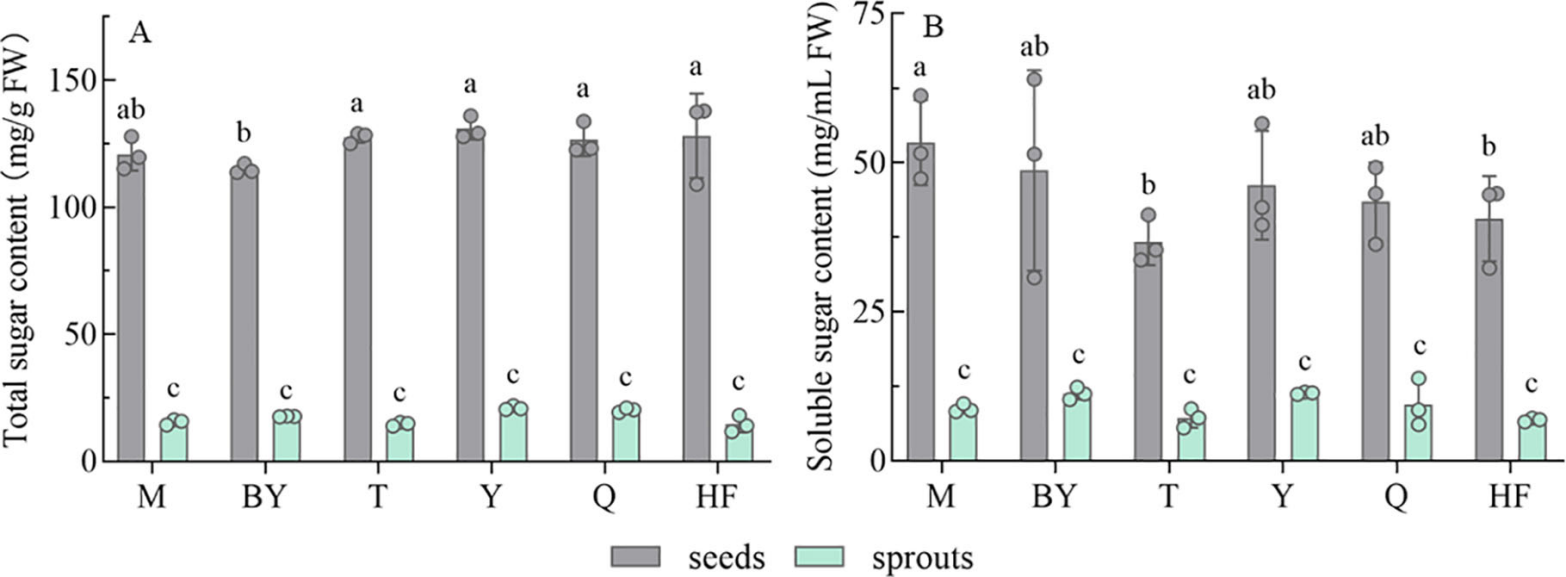
The total and soluble sugar content of seeds and sprouts (germination for 7 days) of six radish varieties. Total Sugar **(A)**, Soluble Sugar **(B)**; mg/g FW, mg/mL FW indicates the comparison of the content of the target substance per gram of seed and per gram of fresh sprout. The data represent mean ± SD values (n = 3). The different lowercase letters indicated significant differences in values (P < 0.05).

**Figure 2** shows the determination of the total and soluble sugar contents in seeds and corresponding sprouts of six radish varieties, essential nutrients in food. Both contents were significantly higher in all seeds than in sprouts. Total sugar content in seeds ranged from 115.05 to 130.99 mg/g FW, with the highest in Y and the lowest in BY. The content in sprouts ranged from 14.65 to 21.06 mg/g FW, and the overall difference in content was not significant (**Figure 2A**). Soluble sugar content in seeds ranged from 36.78 to 53.37 mg/mL FW, with M being the highest and T the lowest; and in sprouts ranged from 14.65 to 20.22 mg/mL FW (**Figure 2B**).

### 3.3 VC content

**Figure 3:**
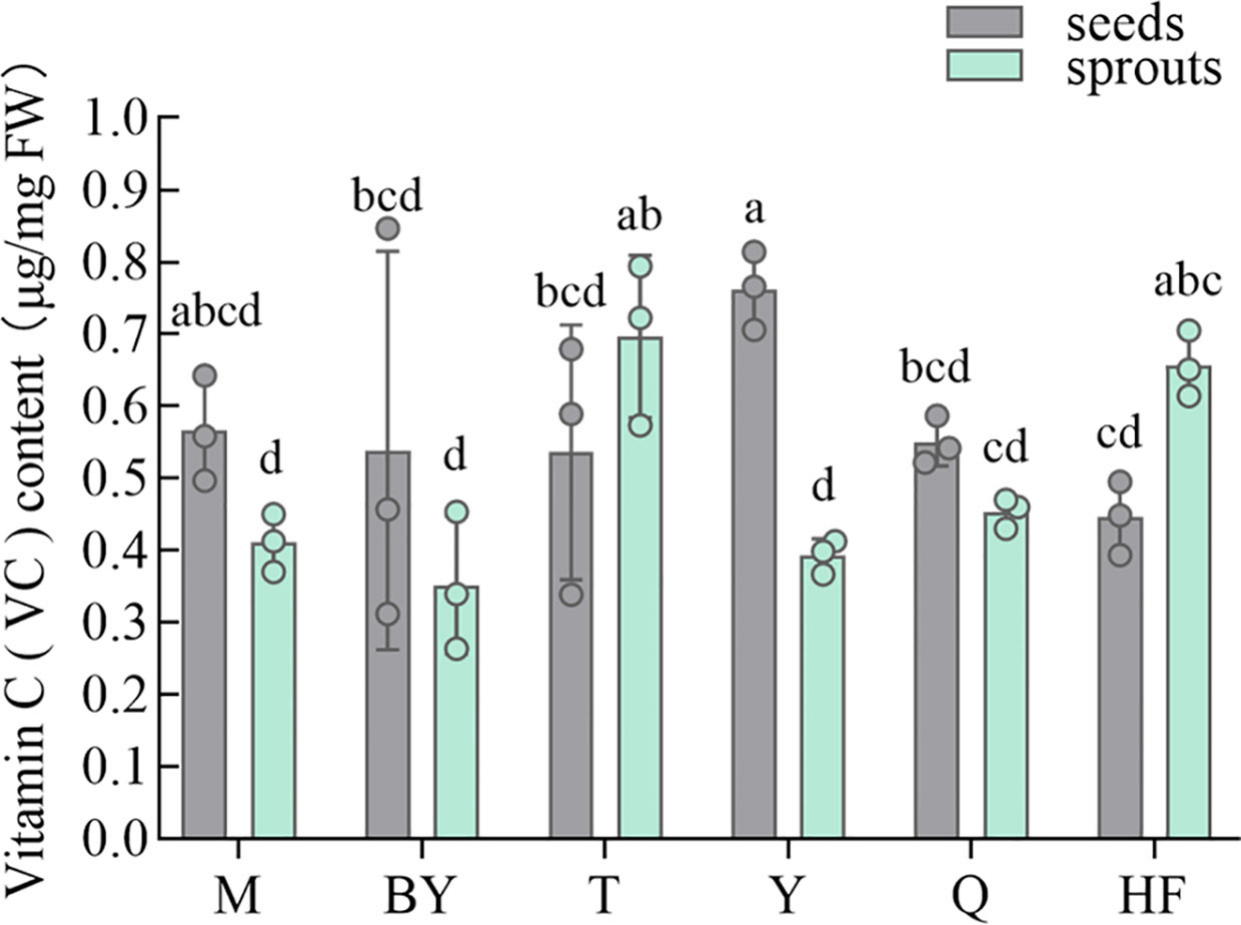
Vitamin C content of seeds and sprouts (germination for 7 days) of six radish varieties. The data represent mean ± SD values (n = 3). The different lowercase letters indicated significant differences in values (P < 0.05).

In **Figure 3**, VC content of seeds and sprouts of different radish varieties varied considerably. With the germination of seeds, the concentration of VC decreased in M, BY, Y, and Q, but increased in T and HF. T, Y, and HF seeds and sprouts differed significantly in their VC content. In seeds, the highest content of VC was Y (0.76 μg/mg FW), the content of the six varieties ranged from 0.44 to 0.76 μg/mg FW. The highest concentration was T in sprouts (0.70 μg/mg FW), ranging from 0.35 to 0.70 μg/mg FW.

### 3.4 TP content

**Figure 4:**
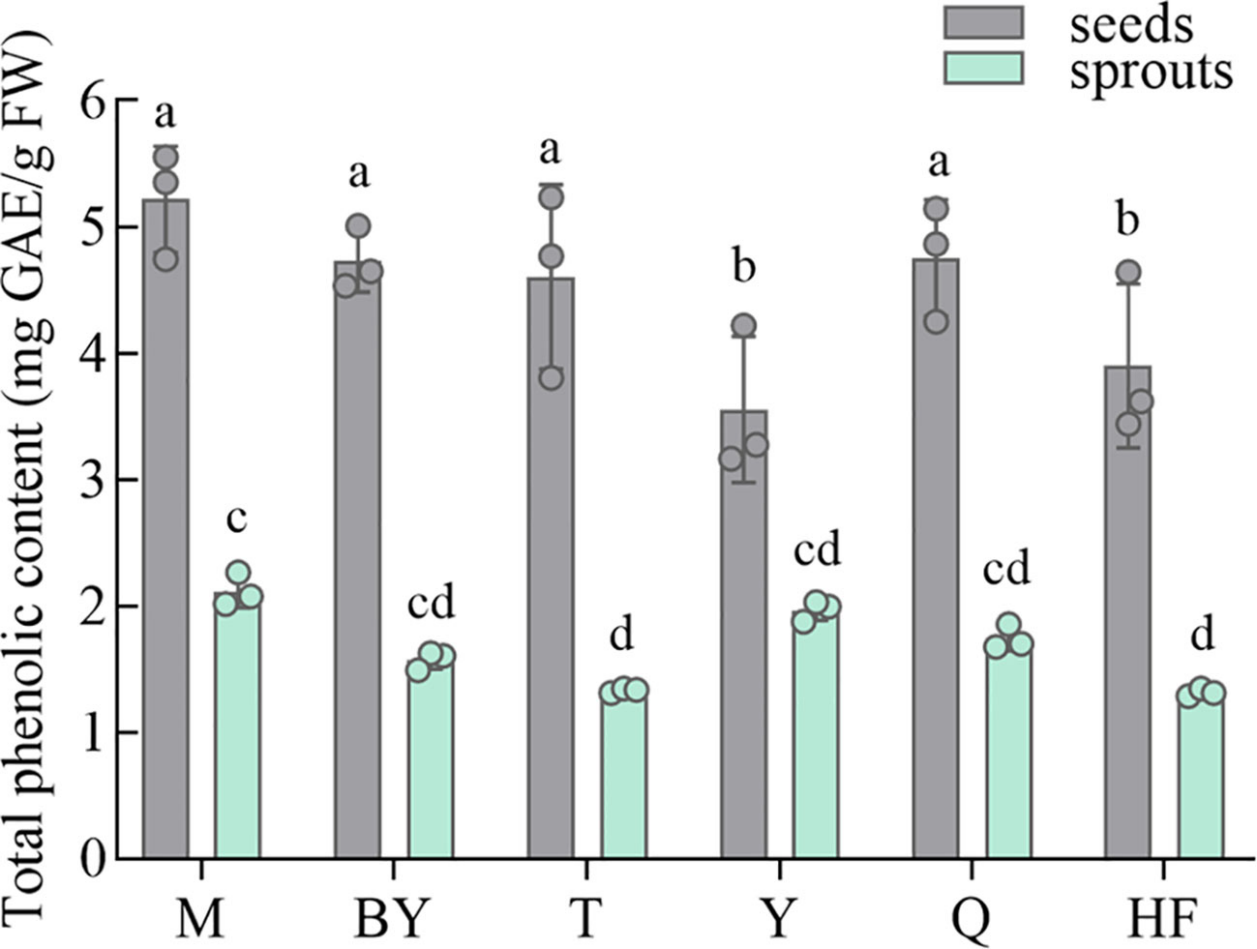
Total phenolic content of six radish seeds and seven-day-old sprouts. The data represent mean ± SD values (n = 3). The different lowercase letters indicated significant differences in values (P < 0.05).

In **Figure 4**, the content of TP content in seeds (3.55 to 5.22 mg GAE/g FW) was significantly higher than that in sprouts (1.31 to 2.12 mg GAE/g FW). Decrease in content was measured to be about 1.8 to 3.5-fold. The highest TP content was found in M seeds followed by Q, BY, and T, which were significantly higher than HF and Y. No obvious difference was noticed in TP content among sprouts of the six varieties.

### 3.5 The content of GLSs

**Figure 5:**
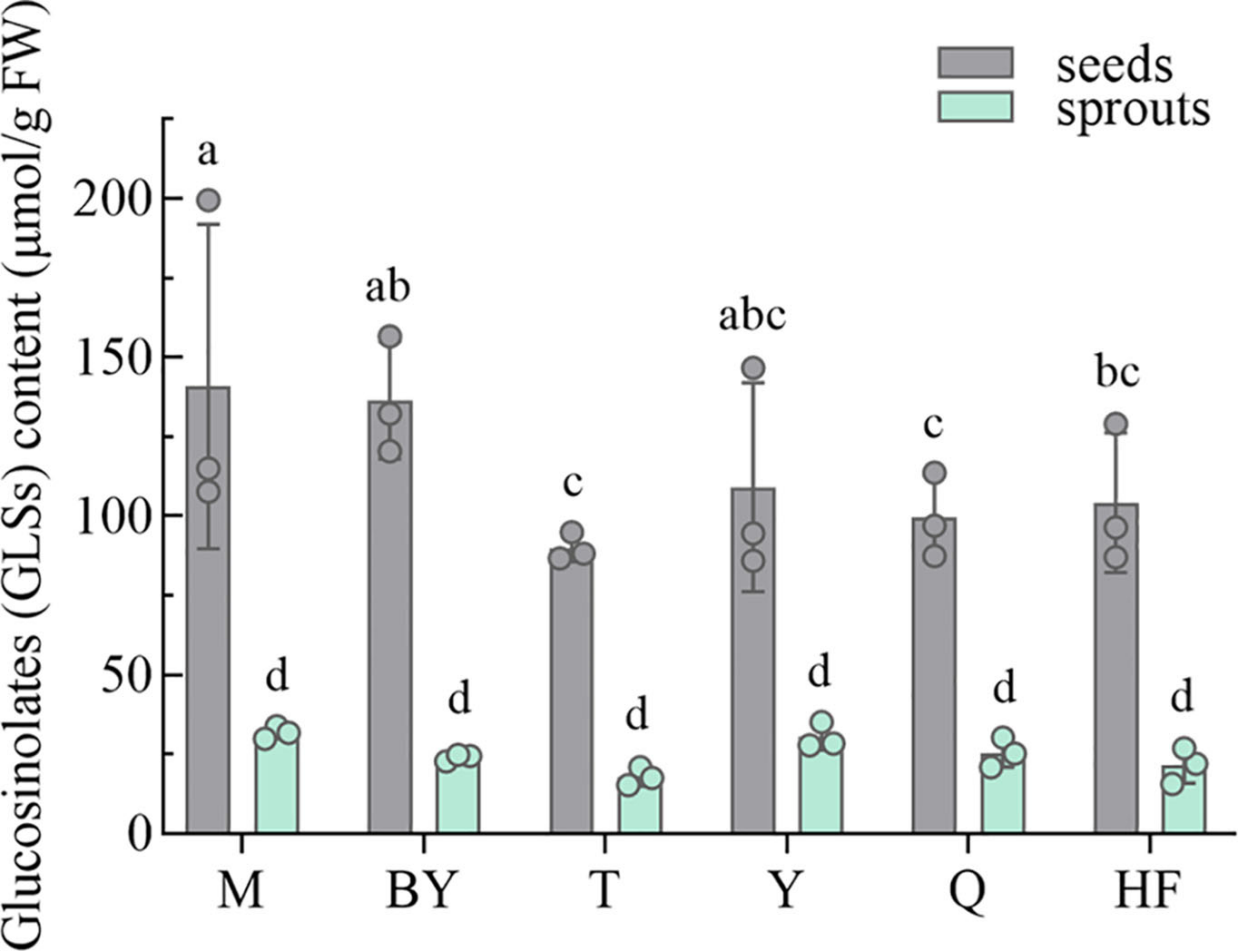
Glucosinolates (GLSs) content of six radish seeds and their seven-day sprouts. The data represent mean ± SD values (n = 3). The different lowercase letters indicated significant differences in values (P < 0.05).

GLSs in seeds and sprouts of six radish varieties were compared and are presented in **Figure 5**. Based on fresh weight, significantly higher levels of GLSs were found in radish seeds than in sprouts, showing inter-varietal differences. Concentrations of GLSs in seeds were about 3 to 6-fold higher than in sprouts. M (140.70 μmol/g FW) and BY (136.38 μmol/g FW) seeds had the highest levels of GLSs, followed by Y (108.98 μmol/g FW) and HF (104.14 μmol/g FW), and the lowest levels of Q (99.32 μmol/g FW) and T (89.84 μmol/g FW). The yield of GLSs from sprouts decreased dramatically from 21.49 to 31.88 μmol/g FW during germination for six radish varieties, with considerable inter-varietal differences.

### 3.6 Antioxidant capacities

As shown in **Table 1**, three different antioxidants were used to evaluate the antioxidant capacity. The results obtained under different methods were different. Much higher POD activity of radish sprouts was measured than that of seeds (p<0.05). POD content was significantly higher in Y sprouts than in several other species, followed by M, T, BY, and finally Q and HF. HF had the highest POD content in seeds, but the differences in the contents among species were not significant. Compared with seeds, there was little difference in PAL activity in sprouts (p>0.05). The same results were obtained between different varieties. T-AOC of seeds was significantly higher than that of sprouts (p<0.05), with the strongest T-AOC (30.76 U/g FW) found in M among different varieties.

**Table 1:** Antioxidant activity of radish seeds and sprouts.

Cultivar	POD (U/g FW)		PAL (U/g FW)		T-AOC (U/g FW)	
	Seeds	Sprouts	Seeds	Sprouts	Seeds	Sprouts
M	14.73 ± 2.31e	471.21 ± 53.70b	160.60 ± 10.72a	151.82 ± 7.20a	30.76 ± 3.29a	7.21 ± 0.44e
BY	17.54 ± 0.36e	325.44 ± 43.42c	167.71 ± 22.24a	150.70 ± 18.84a	20.42 ± 0.99d	6.27 ± 0.81e
T	14.15 ± 8.25e	410.53 ± 42.68bc	168.06 ± 20.33a	160.03 ± 4.75a	20.20 ± 0.49d	5.51 ± 0.58e
Y	19.07 ± 5.25e	591.19 ± 142.61a	178.69 ± 11.33a	150.50 ± 24.99a	27.89 ± 1.94b	7.53 ± 0.33e
Q	12.25 ± 1.23e	191.60 ± 32.89d	176.44 ± 19.77a	178.20 ± 17.06a	23.47 ± 0.99c	7.60 ± 0.91e
HF	33.40 ± 0.93e	166.51 ± 24.79d	176.90 ± 10.22a	166.89 ± 22.90a	20.06 ± 0.95d	6.15 ± 0.25e

^I^Within columns, values (n=3; ± SD) with different lowercase letters indicate significant differences at P<0.05 according to Duncan’s test. POD, Peroxidase; PAL, Phenylalanine ammonia lyase; T-AOC, Total antioxidant capacity.

### 3.7 Optimal variety determination based on the TOPSIS-entropy weight method

The current analysis was used to comprehensively evaluate and analyze seeds and sprouts samples from six different radish varieties, enabling the confirmation of the optimal radish variety. The selection was determined based on chlorophyll, carotenoid, anthocyanin, total sugars, soluble sugars, VC, TP, GLSs, POD, PAL, and T-AOC. All criteria were considered positive. In **Table 2**, chlorophyll *a* had the largest weight and the greatest influence on the comprehensive evaluation of radish varieties, followed by POD and chlorophyll *b*. VC and PAL had the smallest weights and thus had little influence. **Table 3** shows the final selection ranking of radish varieties obtained by TOPSIS in combination with different assays. A higher Ci value may reveal a higher combined value of major phytochemicals and bioactivities. The combined ranking was M seeds > M sprouts > Y sprouts > T sprouts > Q sprouts > BY sprouts > HF sprouts > Y seeds > Q seeds > BY seeds > T seeds > HF seeds. Altogether, M might be the optimal radish variety.

**Table 2:** Weighting of phytochemicals and biological activities.

Categories	Weight	Number
Chlorophyll A	0.1076	1
Chlorophyll B	0.1031	3
Carotenoid	0.0786	8
Anthocyanin	0.0994	4
Total sugar	0.0979	5
Soluble sugar	0.0834	6
Vitamin C (VC)	0.0435	12
Total phenolic (TP)	0.0696	10
Glucosinolates (GLSs)	0.0778	9
POD	0.1042	2
PAL	0.0524	11
T-AOC	0.0825	7

**Table 3:** Final ranking of radish varieties under different phytochemical contents and bioactivities by TOPSIS.

Cultivar	L^-^	L^+^	C_i_	Rank
Sprouts				
M	0.0876	0.0919	0.4881	2
BY	0.0707	0.1132	0.3845	6
T	0.0895	0.1095	0.4498	4
Y	0.0865	0.0939	0.4795	3
Q	0.0913	0.1124	0.4482	5
HF	0.0609	0.1157	0.3449	7
Seeds				
M	0.1070	0.1064	0.5014	1
BY	0.0621	0.1323	0.3194	10
T	0.0561	0.1274	0.3058	11
Y	0.0644	0.1279	0.3349	8
Q	0.0603	0.1272	0.3217	9
HF	0.0560	0.1310	0.2997	12

L^+^ is the distance between each evaluation index and the positive ideal solution, L^-^ refers to the distance between each evaluation index and the negative ideal solution, and C_i_ represents the relative proximity.

## 4 Discussion

Chlorophyll, carotenoid, and anthocyanin jointly participate in the production of various colors of leaves, flowers, and fruits of plants (Liu et al., 2021). Chlorophylls play an essential role in the photosynthesis of plants. The main type is chlorophyll *a*, presenting blue-green under light conditions, and chlorophyll *b*, forming yellow-green, at a distribution ratio of about 3:1 (Gebregziabher et al., 2021). Chlorophylls and their derivatives positively affect human health through anti-mutation, anti-cancer, and anti-inflammatory activities (Ferruzzi and Blakeslee, 2007). While carotenoids are widely known as pro-vitamin A and are important in reducing the risk of eye diseases such as cataracts and age-related macular degeneration, as well as maintaining heart health and reducing ultraviolet light-induced skin damage (Eggersdorfer and Wyss, 2018). Anthocyanins are widely found in plant vacuoles and also act as antioxidants. Anthocyanins also have the potential to treat cardiovascular diseases such as hypertension and atherosclerosis, besides the function of eliminating free radicals in plants (Garcia and Blesso, 2021). These three components could be used to reveal the quality of radish microgreens. Pigmentation is considered an important parameter in determining the quality of sprouts in view of its powerful roles in living plants, in addition to assisting photosynthesis based on light capture. Still, the color also influences the consumer’s choice and acceptance preference (Tilahun et al., 2023). Previously, the total chlorophyll (chlorophyll *a* + *b*) content of radish sprouts harvested for 10 days was determined to be in the range of 0.30 to 0.60 mg/g DW (Tilahun et al., 2023). Here, we obtained a total chlorophyll content of 0.264 mg/g FW for the highest variety of radish sprouts, which was comparable to but slightly lower than that reported previously, and this may be related to differences in radish varieties (Wojdyło et al., 2020; Kyriacou et al., 2019). Chlorophyll content in various sprouts (e.g. Radish, Broccoli, Tatsoi, Cabbage, Mustard, etc.) range from 0.014 to 1.842 mg/g FW (Kyriacou et al., 2019; Wojdyło et al., 2020). Carotenoid levels exhibited the same pattern as chlorophyll levels. Previous studies have reported similar levels of various carotenoids (0.022-0.949 mg/g FW) (Wojdyło et al., 2020).

Energy is a major prerequisite for plant growth. Cell division and differentiation in plants depend on sugars to supply nutrients and signaling molecules. Sugars can also scavenge free radicals, alter cell osmotic pressure and enhance stress resistance. Soluble sugar may act as a major supplier of nutrients, further boosting the generation of macromolecules and energy, as well as specific and coordinated development (Eveland and Jackson, 2012; Sami et al., 2019). Seeds can retain a high percentage of energy compounds, including sugars (Zhao et al., 2020). Similar to previous studies (Wojdyło et al., 2020; Kyriacou et al., 2019), our study revealed that sugar contents did not vary excessively according to radish variety. Germination of seeds, utilizing its storage material, significantly reduced the total and soluble sugar content, which was consistent with prior report (Alipor et al., 2019). Their contents are comparable to other fruits or vegetables, such as broccoli and sunflower sprouts, respectively (Wojdyło et al., 2020).

VC can efficiently scavenge oxidative stress and retard reactive oxygen species production in response to abiotic stresses. Humans need to eat a lot of VC, rather than other vitamins, to maintain health, with the effect of preventing anemia, cancer, etc (Fujii, 2021; Noreen et al., 2021). Our detection of VC content in radish sprouts were similar to the previously reported data (Kyriacou et al., 2019; Šola et al., 2024; Tilahun et al., 2023). There was heterogeneous pattern of VC in seeds and sprouts of different radish varieties, largely due to the variety (Pajak et al., 2014).

Foods rich in polyphenols can be consumed to benefit the health preservation for patients with chronic diseases, such as cardiovascular disease, cancer, diabetes and so on, even to a lesser extent positively impacting human brain function (Fraga et al., 2019). Vegetables and cereals, including both seeds and sprouts, are good sources of phenolic compounds, and radishes offer a large proportion of phenolic acid (Pajak et al., 2014). Our result was consistent with several publications (Baenas et al., 2012; Lv et al., 2020). However, more studies have confirmed sprouts’ higher taxonomic content than corresponding seeds (Gawlik-Dziki et al., 2016; Chen et al., 2017; Francis et al., 2022). It is impossible to explain this difference, which may be influenced by various factors such as plant type, assay method, etc. It is unclear how phenolic levels change throughout the germination process, which may be caused by the complex biochemical metabolism of seeds (Dueñas et al., 2009). This also needs to be explored in the next research. Similarly, Tilahun et al. (2023) analyzed five varieties of radish sprouts with TP content ranging from 1.5 to 3.0 mg GAE/g DW. Francis et al. (2022) reported polyphenol content of radish seeds ranging from 2 to 4 mg GAE/g DW. Previously reported ranges and trends of TP content in broccoli seeds and sprouts were consistent with our results (Lv et al., 2020). The polyphenol contents of radish seeds and sprouts were also comparable to other fruits or vegetables, such as apples, red grapes, strawberries, peaches, bananas, red onions, spinach, and peppers, respectively (Lin and Tang, 2007). Altogether, these discoveries support the health benefits of radish seeds and sprouts.

GLSs, unique among cruciferous plants, are precursor compounds of isothiocyanates (ITCs) with definite functional properties (Alloggia et al., 2023), and the main reason for their consumption recommendation. GLSs represent an important class of secondary plant products, are unique to Brassicaceae, which are the precursor compounds to ITCs. It has antioxidant, antibacterial, anti-inflammatory, and other active functions, can reduce the incidence and severity of many degenerative diseases (*e.g.*, cardiovascular disease, metabolic disorder and tumors) (Chartoumpekis et al., 2019). It was reported that the content of GLSs decreased gradually during the growth of sprouts, and was significantly higher than that in mature vegetables (Pérez-Balibrea et al., 2008). To optimize bioactive component-rich fresh foods to maintain healthy, the key lies in the selection of plants with the optimal phytochemical composition, including seeds and sprouts (Baenas et al., 2017). Given that radishes belong to the genus brassica as well as the edible and medical properties of their seeds, radish seeds and sprouts are excellent raw materials with health benefits. Concentrations of GLSs in seeds were about 3 to 6-fold higher than in sprouts, in agreement with those recorded by Baenas et al. (2014). The content of GLSs (100 to 140 μmol/g DW) in 7-day-old radish sprouts was close to the measured value in seeds by Bowen-Forbes et al. (2023). While different levels of GLSs in sprouts were also reported separately (Baenas et al., 2014; Bowen-Forbes et al., 2023; Hanlon and Barnes, 2011). Its content in radish seeds ranged from 167 to 1052 mg/100 g FW in previous study (Baenas et al., 2014). Consistently, our research revealed that the content of GLSs was highest at the seed stage and decreased with increasing days of growth in cruciferous plants (Baenas et al., 2012; Gamba et al., 2021).

POD is an important metabolic enzyme in the growth stage of plants. It can modulate the plant antioxidant system by differentiation, seed germination, fruit maturation, and aging. Our early research has generated similar values in POD content (Zhao and Zhu, 2014). PAL, an important enzyme in plant secondary metabolism, is involved in the synthesis of lignin, flavonoids, and coumarin, exhibiting potential in human disease management (Levy et al., 2018; Kawatra et al., 2020). T-AOC of radish depends on the type and content of antioxidant, which may be higher in the case of greater variety and content (Zhao and Zhu, 2014). T-AOC of Brassicaceae sprouts was found to depend mainly on anthocyanins (Steyn et al., 2002), phenols (Kim et al., 2006), and GLSs (Williamson et al., 1998). The content of TP and GLSs in radish seed was significantly higher than that of sprout in this experiment. It can be inferred that the decrease in T-AOC during radish germination would be explained by the decrease in the content of these substances.

TOPSIS method is one of the main functional feature of this paper, which is a multi-objective decision-making method. The information entropy is generally utilized to understand the degree of dispersion of a certain indicator. A smaller value may suggest a greater degree of dispersion, and thus a stronger influence (i.e., weight) on the comprehensive evaluation. The indicator may occupy none obvious position in the evaluation if all the values of a certain indicator are equal. Therefore, acting as a tool for weight calculation of each indicator, the information entropy may offer interpretable data for the comprehensive evaluation of multiple indicators. TOPSIS comprehensive evaluation, i.e., a way to rank following the proximity of a finite number of evaluation objects to the idealized target, is the evaluation of the relative merits and demerits of the existing objects. It is a kind of ranking approaching to the ideal solution, which is frequently employed with definite value in the analysis of multi-objective decision-making. It is also known as the superiority and inferiority solution distance method (Ansarifar et al., 2015; Khodaei et al., 2021). In our exploration, the variety M exhibited the highest ranking of seeds and sprouts, which is validated to be the optimal radish variety. While seeds of the other five varieties were rated lower than sprouts. Hence, the combined value of phytochemicals and bioactivities of sprouts was generally higher than that of the seeds. As a result, consumption of radish sprouts, with superior chemical composition, is more recommended for health promotion in daily life, without need to consider the variety. Of course, it is also recommended to consume radish seeds, for its higher content of GLSs, for different selection needs.

## 5 Conclusions

In the present research, genotype is speculated to be the main factor influencing their values in radish seeds and sprouts, taking into consideration of change trends of phytochemical compounds we investigated. Seeds have higher content of GLSs, TP, total sugars, and soluble sugars than sprouts. While sprouts possess higher chlorophyll, carotenoids, and POD content than seeds. The contents of GLSs, chlorophyll, carotenoids, anthocyanins, VC, TP, POD, and T-AOC varied greatly among different varieties. Therefore, it is necessary to obtain the optimum radish variety by using suitable tools for comprehensive evaluation. Our selection of M as the optimal variety has achieved by comprehensive evaluation based on TOPSIS-entropy weight method. Moreover, sprouts outperform seeds, which is hence recommended to consume more in daily life to obtain more bioactive components. For more GLSs, it is recommended to consume radish seeds. Currently, radish seeds may not be commonly consumed due to insufficient public awareness of their health properties and the consumption method. Our data supports it as a good source of health-promoting functional foods and that it can be consumed similarly to other edible seed classes, in salads, in soups or directly. Our experiments on radish seeds also aim to increase consumers’ knowledge, thus expanding the range of functional food choices and allowing consumers to choose radish seeds according to their needs based on the difference in phytochemical content between radish and seedling phytoconstituents. While some shortcomings should be emphasized in our study: (1) It is still not clear whether the levels of phytochemicals and antioxidants obtained directly from seeds are higher than in sprouts germinated from the same number of seeds, despite comparisons made between radish seeds and sprouts. (2) The range of radish varieties, with 6 types merely at this time, can be further expanded in subsequent studies. (3) We only detected the amount of GLSs in radishes, and subsequent experiments can further examine the amount of its metabolite ITCs. Considering an increased level of affection for edible plants, this study significantly improves our understanding of the nutritional profile of radish seeds and sprouts, revealing them to be a good source of health-promoting bioactive compounds. Sprouts or seeds of recommended radish varieties be consumed daily as superfoods or functional foods. Data generated here may also guide cultivation practices to improve radishes’ phytochemical compositions.

## Data Availability

The raw data supporting the conclusions of this article will be made available by the authors, without undue reservation.

## References

[B1] Alipor S. Taghvaei M. Jalilian A. Kazemeini A. Razi H. (2019). Hydro-thermal priming enhance seed germination capacity and seedling growth in sugar beet. Cell Mol. Biol. (Noisy-le-grand) 65, 90–96. doi: 10.14715/cmb/2019.65.4.15 31078157

[B2] Alloggia F. P. Bafumo R. F. Ramirez D. A. Maza M. A. Camargo A. B. (2023). Brassicaceae microgreens: A novel and promissory source of sustainable bioactive compounds. Curr. Res. Food Sci. 6, 100480. doi: 10.1016/j.crfs.2023.100480 36969565 PMC10030908

[B3] Ansarifar E. Shahidi F. Mohebbi M. Razavi S. M. Ansarifar J. (2015). A new technique to evaluate the effect of chitosan on properties of deep-fried Kurdish cheese nuggets by TOPSIS. LWT - Food Sci. Technol. 62, 1211–1219. doi: 10.1016/j.lwt.2015.01.051

[B4] Aruna G. Yerragunt V. G. Raju A. (2012).Photochemistry and pharmacology ofraphanussativus. Available online at: https://www.semanticscholar.org/paper/PHOTOCHEMISTRY-AND-PHARMACOLOGY-OFRAPHANUSSATIVUS-Aruna-Yerragunt/406d849b07a8dd9882ae55d27235d2b434b50405 (Accessed November 19, 2024).

[B5] Baenas N. García-Viguera C. Moreno D. A. (2014). Biotic elicitors effectively increase the glucosinolates content in Brassicaceae sprouts. J. Agric. Food Chem. 62, 1881–1889. doi: 10.1021/jf404876z 24484436

[B6] Baenas N. Gómez-Jodar I. Moreno D. A. García-Viguera C. Periago P. M. (2017). Broccoli and radish sprouts are safe and rich in bioactive phytochemicals. Postharvest Biol. Technol. 127, 60–67. doi: 10.1016/j.postharvbio.2017.01.010

[B7] Baenas N. Moreno D. A. García-Viguera C. (2012). Selecting sprouts of brassicaceae for optimum phytochemical composition. J. Agric. Food Chem. 60, 11409–11420. doi: 10.1021/jf302863c 23061899

[B8] Bowen-Forbes C. Armstrong E. Moses A. Fahlman R. Koosha H. Yager J. Y. (2023). Broccoli, kale, and radish sprouts: key phytochemical constituents and DPPH free radical scavenging activity. Molecules 28, 4266. doi: 10.3390/molecules28114266 37298743 PMC10254352

[B9] Chartoumpekis D. V. Ziros P. G. Chen J.-G. Groopman J. D. Kensler T. W. Sykiotis G. P. (2019). Broccoli sprout beverage is safe for thyroid hormonal and autoimmune status: Results of a 12-week randomized trial. Food Chem. Toxicol. 126, 1–6. doi: 10.1016/j.fct.2019.02.004 30735751 PMC6422739

[B10] Chen J. Wang X. (2006). Guide to experiments in plant physiology (Guangzhou: South China University of Technology Press).

[B11] Chen Z. Wang P. Weng Y. Ma Y. Gu Z. Yang R. (2017). Comparison of phenolic profiles, antioxidant capacity and relevant enzyme activity of different Chinese wheat varieties during germination. Food Biosci. 20, 159–167. doi: 10.1016/j.fbio.2017.10.004

[B12] Choe U. Yu L. L. Wang T. T. Y. (2018). The science behind microgreens as an exciting new food for the 21st century. J. Agric. Food Chem. 66, 11519–11530. doi: 10.1021/acs.jafc.8b03096 30343573

[B13] Dueñas M. Hernández T. Estrella I. Fernández D. (2009). Germination as a process to increase the polyphenol content and antioxidant activity of lupin seeds (Lupinus angustifolius L.). Food Chem. 117, 599–607. doi: 10.1016/j.foodchem.2009.04.051

[B14] Eggersdorfer M. Wyss A. (2018). Carotenoids in human nutrition and health. Arch. Biochem. Biophysics 652, 18–26. doi: 10.1016/j.abb.2018.06.001 29885291

[B15] Eveland A. L. Jackson D. P. (2012). Sugars, signaling, and plant development. J. Exp. Bot. 63, 3367–3377. doi: 10.1093/jxb/err379 22140246

[B16] Ferruzzi M. G. Blakeslee J. (2007). Digestion, absorption, and cancer preventative activity of dietary chlorophyll derivatives. Nutr. Res. 27, 1–12. doi: 10.1016/j.nutres.2006.12.003

[B17] Fraga C. G. Croft K. D. Kennedy D. O. Tomás-Barberán F. A. (2019). The effects of polyphenols and other bioactives on human health. Food Funct. 10, 514–528. doi: 10.1039/c8fo01997e 30746536

[B18] Francis H. Debs E. Koubaa M. Alrayess Z. Maroun R. G. Louka N. (2022). Sprouts Use as Functional Foods. Optimization of Germination of Wheat (Triticum aestivum L.), Alfalfa (Medicago sativa L.), and Radish (Raphanus sativus L.) Seeds Based on Their Nutritional Content Evolution. Foods 11, 1460. doi: 10.3390/foods11101460 35627030 PMC9141080

[B19] Fujii J. (2021). Ascorbate is a multifunctional micronutrient whose synthesis is lacking in primates. J. Clin. Biochem. Nutr. 69, 1–15. doi: 10.3164/jcbn.20-181 34376908 PMC8325764

[B20] Gamba M. Asllanaj E. Raguindin P. F. Glisic M. Franco O. H. Minder B.. (2021). Nutritional and phytochemical characterization of radish (*Raphanus sativus*): A systematic review. Trends Food Sci. Technol. 113, 205–218. doi: 10.1016/j.tifs.2021.04.045

[B21] Gan R.-Y. Lui W.-Y. Wu K. Chan C.-L. Dai S.-H. Sui Z.-Q.. (2017). Bioactive compounds and bioactivities of germinated edible seeds and sprouts: An updated review. Trends Food Sci. Technol. 59, 1–14. doi: 10.1016/j.tifs.2016.11.010

[B22] Garcia C. Blesso C. N. (2021). Antioxidant properties of anthocyanins and their mechanism of action in atherosclerosis. Free Radical Biol. Med. 172, 152–166. doi: 10.1016/j.freeradbiomed.2021.05.040 34087429

[B23] Gawlik-Dziki U. Dziki D. Nowak R. Świeca M. Olech M. Pietrzak W. (2016). Influence of sprouting and elicitation on phenolic acids profile and antioxidant activity of wheat seedlings. J. Cereal Sci. 70, 221–228. doi: 10.1016/j.jcs.2016.06.011

[B24] Gebregziabher B. S. Zhang S. Qi J. Azam M. Ghosh S. Feng Y.. (2021). Simultaneous determination of carotenoids and chlorophylls by the HPLC-UV-VIS method in soybean seeds. Agronomy 11, 758. doi: 10.3390/agronomy11040758

[B25] Hanlon P. R. Barnes D. M. (2011). Phytochemical composition and biological activity of 8 varieties of radish (Raphanus sativus L.) sprouts and mature taproots. J. Food Sci. 76, C185–C192. doi: 10.1111/j.1750-3841.2010.01972.x 21535648

[B26] Jacky C. (2023).Chinese Pharmacopoeia 2020 - English ed. issued in March 2023. In: CISEMA - China Zertifizierung, Einkauf und Qualitätssicherung. Available online at: https://www.cisema.com/en/chinese-pharmacopoeia-2020-edition-official-english-translation/ (Accessed November 19, 2024).

[B27] Kakkar S. Tandon R. Tandon N. (2023). The rising status of edible seeds in lifestyle related diseases: A review. Food Chem. 402, 134220. doi: 10.1016/j.foodchem.2022.134220 36137389

[B28] Kawatra A. Dhankhar R. Mohanty A. Gulati P. (2020). Biomedical applications of microbial phenylalanine ammonia lyase: Current status and future prospects. Biochimie 177, 142–152. doi: 10.1016/j.biochi.2020.08.009 32828824

[B29] Khodaei D. Hamidi-Esfahani Z. Rahmati E. (2021). Effect of edible coatings on the shelf-life of fresh strawberries: A comparative study using TOPSIS-Shannon entropy method. NFS J. 23, 17–23. doi: 10.1016/j.nfs.2021.02.003

[B30] Kim H.-J. Chen F. Wang X. Choi J.-H. (2006). Effect of methyl jasmonate on phenolics, isothiocyanate, and metabolic enzymes in radish sprout (Raphanus sativus L.). J. Agric. Food Chem. 54, 7263–7269. doi: 10.1021/jf060568c 16968092

[B31] Kyriacou M. C. El-Nakhel C. Graziani G. Pannico A. Soteriou G. A. Giordano M.. (2019). Functional quality in novel food sources: Genotypic variation in the nutritive and phytochemical composition of thirteen microgreens species. Food Chem. 277, 107–118. doi: 10.1016/j.foodchem.2018.10.098 30502125

[B32] Le T. N. Luong H. Q. Li H.-P. Chiu C.-H. Hsieh P.-C. (2019). Broccoli (Brassica oleracea L. var. italica) Sprouts as the Potential Food Source for Bioactive Properties: A Comprehensive Study on *In Vitro* Disease Models. Foods 8, 532. doi: 10.3390/foods8110532 31671614 PMC6915343

[B33] Levy H. L. Sarkissian C. N. Scriver C. R. (2018). Phenylalanine ammonia lyase (PAL): From discovery to enzyme substitution therapy for phenylketonuria. Mol. Genet. Metab. 124, 223–229. doi: 10.1016/j.ymgme.2018.06.002 29941359

[B34] Li N. Wu X. Zhuang W. Wu C. Rao Z. Du L.. (2022). Cruciferous vegetable and isothiocyanate intake and multiple health outcomes. Food Chem. 375, 131816. doi: 10.1016/j.foodchem.2021.131816 34929422

[B35] Li R. Zhu Y. (2018). The primary active components, antioxidant properties, and differential metabolite profiles of radish sprouts (Raphanus sativus L.) upon domestic storage: analysis of nutritional quality. J. Sci. Food Agric. 98, 5853–5860. doi: 10.1002/jsfa.9137 29786832

[B36] Lin J.-Y. Tang C.-Y. (2007). Determination of total phenolic and flavonoid contents in selected fruits and vegetables, as well as their stimulatory effects on mouse splenocyte proliferation. Food Chem. 101, 140–147. doi: 10.1016/j.foodchem.2006.01.014

[B37] Liu Y. Feng X. Zhang Y. Zhou F. Zhu P. (2021). Simultaneous changes in anthocyanin, chlorophyll, and carotenoid contents produce green variegation in pink-leaved ornamental kale. BMC Genomics 22, 455. doi: 10.1186/s12864-021-07785-x 34139990 PMC8212504

[B38] Liu H.-Y. Liu Y. Li M.-Y. Ge Y.-Y. Geng F. He X.-Q.. (2022). Antioxidant capacity, phytochemical profiles, and phenolic metabolomics of selected edible seeds and their sprouts. Front. Nutr. 9. doi: 10.3389/fnut.2022.1067597 PMC9798843 36590202

[B39] Lv X. Meng G. Li W. Fan D. Wang X. Espinoza-Pinochet C. A.. (2020). Sulforaphane and its antioxidative effects in broccoli seeds and sprouts of different cultivars. Food Chem. 316, 126216. doi: 10.1016/j.foodchem.2020.126216 32044707

[B40] Manivannan A. Kim J.-H. Kim D.-S. Lee E.-S. Lee H.-E. (2019). Deciphering the nutraceutical potential of Raphanus sativus—A comprehensive overview. Nutrients 11, 402. doi: 10.3390/nu11020402 30769862 PMC6412475

[B41] Moreno D. A. Pérez-Balibrea S. García-Viguera C. (2006). Phytochemical quality and bioactivity of edible sprouts. Natural Product Commun. 1, 1934578X0600101120. doi: 10.1177/1934578X0600101120

[B42] Nandi S. Guha P. (2023). Technique for order preference by similarity to ideal solution (TOPSIS): a MCDM approach for selecting suitable solvent considering biochemical profiles and *in vitro* antibacterial efficacy of petioles of betel leaf (Piper betle L.). Environ. Sci. pollut. Res. 30, 46147–46158. doi: 10.1007/s11356-023-25485-9 36715795

[B43] Noreen S. Sultan M. Akhter M. S. Shah K. H. Ummara U. Manzoor H.. (2021). Foliar fertigation of ascorbic acid and zinc improves growth, antioxidant enzyme activity and harvest index in barley (Hordeum vulgare L.) grown under salt stress. Plant Physiol. Biochem. 158, 244–254. doi: 10.1016/j.plaphy.2020.11.007 33221118

[B44] Pajak P. Socha R. Galkowska D. Roznowski J. Fortuna T. (2014). Phenolic profile and antioxidant activity in selected seeds and sprouts. Food Chem. 143, 300–306. doi: 10.1016/j.foodchem.2013.07.064 24054243

[B45] Pérez-Balibrea S. Moreno D. A. García-Viguera C. (2008). Influence of light on health-promoting phytochemicals of broccoli sprouts. J. Sci. Food Agric. 88, 904–910. doi: 10.1002/jsfa.3169

[B46] Šamec D. Pavlović I. Salopek-Sondi B. (2017). White cabbage (Brassica oleracea var. capitata f. alba): botanical, phytochemical and pharmacological overview. Phytochem. Rev. 16, 117–135. doi: 10.1007/s11101-016-9454-4

[B47] Sami F. Siddiqui H. Hayat S. (2019). Interaction of glucose and phytohormone signaling in plants. Plant Physiol. Biochem. 135, 119–126. doi: 10.1016/j.plaphy.2018.11.005 30529977

[B48] Šola I. Vujčić Bok V. Popović M. Gagić S. (2024). Phytochemical composition and functional properties of Brassicaceae microgreens: impact of *in vitro* digestion. Int. J. Mol. Sci. 25, 11831. doi: 10.3390/ijms252111831 39519385 PMC11546364

[B49] Steyn W. J. Wand S. J. E. Holcroft D. M. Jacobs G. (2002). Anthocyanins in vegetative tissues: a proposed unified function in photoprotection. New Phytol. 155, 349–361. doi: 10.1046/j.1469-8137.2002.00482.x 33873306

[B50] Thakur P. Kumar K. Ahmed N. Chauhan D. Eain Hyder Rizvi Q. U. Jan S.. (2021). Effect of soaking and germination treatments on nutritional, anti-nutritional, and bioactive properties of amaranth (Amaranthus hypochondriacus L.), quinoa (Chenopodium quinoa L.), and buckwheat (Fagopyrum esculentum L.). Curr. Res. Food Sci. 4, 917–925. doi: 10.1016/j.crfs.2021.11.019 34927087 PMC8646961

[B51] Tian W. Ehmke L. Miller R. Li Y. (2019). Changes in bread quality, antioxidant activity, and phenolic acid composition of wheats during early-stage germination. J. Food Sci. 84, 457–465. doi: 10.1111/1750-3841.14463 30730580

[B52] Tilahun S. Baek M. W. An K.-S. Choi H. R. Lee J. H. Hong J. S.. (2023). Radish microgreens produced without substrate in a vertical multi-layered growing unit are rich in nutritional metabolites. Front. Plant Sci. 14. doi: 10.3389/fpls.2023.1236055 PMC10536316 37780508

[B53] Williamson G. Faulkner K. Plumb G. W. (1998). Glucosinolates and phenolics as antioxidants from plant foods. Eur. J. Cancer Prev. 7, 17–21. doi: 10.1111/j.1651-2227.2008.01026.x 9511848

[B54] Wojdyło A. Nowicka P. Tkacz K. Turkiewicz I. P. (2020). Sprouts vs. Microgreens as Novel Functional Foods: Variation of Nutritional and Phytochemical Profiles and Their *In vitro* Bioactive Properties. Molecules 25, 4648. doi: 10.3390/molecules25204648 33053861 PMC7587365

[B55] Wunthunyarat W. Seo H.-S. Wang Y.-J. (2020). Effects of germination conditions on enzyme activities and starch hydrolysis of long-grain brown rice in relation to flour properties and bread qualities. J. Food Sci. 85, 349–357. doi: 10.1111/1750-3841.15008 31957892

[B56] Yu X. Cai X. Luo L. Wang J. Ma M. Wang M.. (2020). Influence of tea polyphenol and bovine serum albumin on tea cream formation by multiple spectroscopy methods and molecular docking. Food Chem. 333, 127432. doi: 10.1016/j.foodchem.2020.127432 32659661

[B57] Zhao K. Zhao C. Yang M. Yin D. (2020). ZnCl2 treatment improves nutrient quality and Zn accumulation in peanut seeds and sprouts. Sci. Rep. 10, 2364. doi: 10.1038/s41598-020-59434-0 32047255 PMC7012847

[B58] Zhao X. Zhu Y. (2014). Effect of chitosan treatment on the quality of radish sprouts and their physiological and biochemical properties. North. Hortic. 0, 9–13.

[B59] Zhou C. Zhu Y. Luo Y. (2013). Effects of sulfur fertilization on the accumulation of health-promoting phytochemicals in radish sprouts. J. Agric. Food Chem. 61, 7552–7559. doi: 10.1021/jf402174f 23855586

